# Cross Reactive Cellular Immune Response to HCV Genotype 1 and 4 Antigens among Genotype 4 Exposed Subjects

**DOI:** 10.1371/journal.pone.0101264

**Published:** 2014-06-30

**Authors:** Iman F. Galal, Zainab Zakaria, Walaa R. Allam, Mohamed A. Mahmoud, Ahmed R. Ezzat, Ahmed Osman, Imam Waked, G. Thomas Strickland, Sayed F. Abdelwahab

**Affiliations:** 1 Egyptian Company for Blood Transfusion Services (Egyblood)/VACSERA; Agouza, Giza, Egypt; 2 Department of Hepatology, National Liver Institute, Menoufiya University, Menoufiya, Egypt; 3 Department of Zoology, Faculty of Science, Ain Shams University, Cairo, Egypt; 4 Department of Biochemistry, Faculty of Science, Ain Shams University, Cairo; 5 Department of Epidemiology and Public Health, University of Maryland School of Medicine, Baltimore, Maryland, United States of America; 6 Department of Microbiology and Immunology, Faculty of Medicine, Minia University, Minia, Egypt; Saint Louis University, United States of America

## Abstract

**Background:**

Hepatitis C Virus (HCV) infection is a global health burden particularly in Egypt, where HCV genotype 4a (GT-4a) predominates. The prevention and control of HCV infection will remain a challenge until the development of an effective vaccine that protects against different genotypes. Several HCV GT-1-based vaccines are in different stages of clinical trials, but antigenic differences could make protection against other genotypes problematic. In this regard, data comparing the cell-mediated immune (CMI) response to different HCV genotypes are limited. We aimed to *ex vivo* investigate whether GT-1-based vaccine may protect against HCV GT-4 infections. This was carried out on samples collected from genotype 4 infected/exposed subjects.

**Methods/Principal Findings:**

The CMI responses of 35 subjects; infected with HCV GT-4/or who had spontaneously-resolved the infection and 10 healthy control subjects; to two sets of seven HCV overlapping 15-mer peptide pools derived from both genotypes; and covering most of the viral proteins; were evaluated. This was carried out using an interferon gamma (IFNγ) enzyme-linked immunospot (ELISpot) assay. Peripheral blood mononuclear cells (PBMC) from 17 subjects (48%) responded to at least one peptide pool derived from GT-1b/GT-4a with 13 subjects responding to peptide pools from both genotypes. A strong correlation was found in the responses to both genotypes (*r* = 0.82, *p*<0.001; 95% confidence interval = 0.562–0.933). The average IFNγ total spot forming cells (SFC)/10^6^ PBMC (±SE) from the responding subjects for GT-1b and GT-4a was 216±56 and 199±55, respectively (*p* = 0.833). Also, there were no significant differences between those who cleared their HCV infection or who remained HCV-RNA positive (*p* = 0.8).

**Conclusion/Significance:**

Our data suggest that an effective GT-1b vaccine could protect from GT-4a infection. These data could help in HCV rationale vaccine design and efficacy studies and further our understanding of HCV cross protection against different genotypes.

## Introduction

Hepatitis C virus (HCV) infects almost 3% of the world's population with Egypt having the highest HCV prevalence in the world; 14.7% of the adult population [1]. Egypt has an estimated annual incidence of about 150,000 cases [2], [3]. About 90% of Egyptian HCV isolates belong to HCV genotype 4a; GT-4a [4], [5].

The current treatment regimen for HCV in Egypt consists of a combination therapy with pegylated interferon and ribavirin; a treatment which is expensive, curative in only about half the patients, and has many adverse effects [6]–[8]. The recently developed more efficacious direct acting antiviral drugs will remain too expensive for most patients in Egypt and many parts of the world for the next few years [9]. A preventive and/or therapeutic HCV vaccine remains an overwhelming goal to reduce the burden of this major cause of chronic hepatitis, cirrhosis and hepatocellular carcinoma [10]. HCV isolates are classified into 6 major genotypes and more than 80 subtypes [11]. Recently, a seventh genotype has been characterized [12]. The geographic distribution of HCV genotypes differs considerably. In Europe and the USA, HCV-GT-1a and GT-1b are the commonest subtypes. HCV GT-4 is prevalent in Northern and Equatorial Africa and the Middle East, while GT-5 and GT-6 have been identified in South Africa and Hong Kong, respectively [11]. HCV genotypes differ by at least 30% at the nucleotide level, while subtypes within a single genotype may vary by more than 20% [13]. Thus, efficacy against multiple HCV genotypes is an important consideration for any HCV vaccine. Vaccine candidates that have been developed against HCV GT-1 are being evaluated [14], [15]. However, more information is needed to ascertain whether they will be efficacious in other areas of the world where other HCV genotypes are predominant.

In HCV infection, the humoral immune response usually selects for escape mutants, and often fails to eradicate the infection [16], [17]. T-cell responses appear to have a pivotal role in protection against chronic HCV infection [18], [19]. It is feasible to induce efficient HCV-specific T cells through vaccination with HCV antigens. These antigens in adenoviral vectors have elicited effective and durable T-cell responses in healthy humans [20]; and prevented chronic infection in HCV-infected chimpanzees after challenge with heterologous HCV strains [21]. Vaccine-induced HCV-specific T cells in chimpanzees rapidly expanding after infection and prior to HCV challenge suggest an accelerated memory T cell response [15]. Based on this, induction of HCV-specific T cell responses before exposure to infection may be an efficient strategy to protect from acute and/or chronic HCV infection [14]; and it could be a useful adjuvant in a regimen treating chronic infection [10].

HCV GT-1b vaccines are currently in trials in the USA and Europe [10]. Given that there are genotypic differences between the prevalent genotype in Egypt and many other geographic areas than the one used in these vaccines [14], [15], it seems useful to compare the immune response of Egyptian subjects infected with HCV GT-4 to antigens derived from GT-1b. Herein, we compared the HCV antigen-specific cell mediated immune (CMI) responses among subjects infected with GT-4 by an interferon gamma (IFNγ) enzyme-linked immunospot (ELISpot) assay that utilized overlapping 15mer peptide pools derived from both GT-1b and GT-4a isolates.

## Subjects and Methods

### Study subjects

To evaluate CMI cross reactivity to GT-1 and GT-4 antigens, thirty-five subjects infected with HCV GT-4; identified from a parent study to determine the incidence of HCV infection among 859 healthcare workers (HCW) at the Menoufiya University's National Liver Institute (NLI); were enrolled [2], [5]. In addition, ten healthy individuals with no known exposure to HCV served as a control group (unexposed subjects). Written informed consent was obtained from each patient included in the study and the study protocol conforms to the ethical guidelines of the 1975 Declaration of Helsinki as reflected in a priori approval by the institutional review board of the Menoufiya University National Liver Institute (NLI-IRB 00003413 FWA0000227). The consent form and procedures were approved by the NLI-IRB. Data regarding age, gender, residence, education, job position, past medical history, current symptoms, and potential exposures to blood-borne infections and other risk factors were collected [2], [5]. Blood samples for immunological studies and other laboratory testing were collected. None of the subjects had received interferon-α plus ribavirin treatment for HCV infection prior to participation, and thus, it is assumed that HCV antibody positive RNA negative subjects had spontaneously cleared their infections. The HCV-RNA positive subjects were referred for treatment.

### Detection of HCV infection and other biomarkers

Serum alanine aminotransferase (ALT) levels were measured using routine clinical test kits. Anti-HCV was tested by a third generation enzyme immunoassay (EIA; Murex anti-HCV; version 4.0; USA) according to the manufacturer's instructions. Detection and quantification of HCV-RNA was performed on sera after extraction of RNA using Qiagen viral RNA extraction kit (QIAgen, USA) using a quantitative real-time reverse transcriptase polymerase chain reaction (RT-PCR) via strand-specific AgPath-ID one step assay according to the manufacturer's instructions (Life Technologies Corporation, USA). The assay uses HCV-specific primers, and probes and internal controls. HCV genotyping was conducted by restriction fragment-length polymorphism (RFLP) analysis of the 5′ non-coding region using two sets of restriction endonucleases: *Mva*I/*Hinf*I and *Rsa*I/*Hae*III as previously described [22]. The genotyping data were confirmed by conventional PCR using genotype-specific primers as previously described [5], [23].

### Synthetic HCV peptides and control antigens

Recombinant HCV GT-1b (isolate BK) and GT-4a (isolate ED43) overlapping peptide antigens were obtained from the National Institute of Allergy and Infectious Diseases' Biodefense and Emerging Infections Research Resources and Repository. They were composed of 15 (15mer) and overlapping by 11 amino acids. They were divided into two sets of seven pools each. They were labeled with alphabetical letters as follows: “E2”(92 peptides, aa 372-750) representing the viral envelope protein E2, “F”(78 peptides, aa 1027–1349) comprising the N-terminal half of the NS3 protein, “G”(78 peptides, aa 1339–1661) comprising the remaining half of NS3, “H” (79 peptides; aa1651–1977) comprising the NS4a and NS4b proteins, “I” (111 peptides, aa 1967–2421) comprising NS5a protein, “L” (75 peptides, aa 2411–2721) representing NS5b first half, and “M”(72 peptides, aa 2711–3008) covering the remainder of NS5b protein. Negative control cultures included cells stimulated with culture medium alone but containing the solvent used for the preparation of the peptides (dimethyl sulfoxide, DMSO). Cytomegalovirus lysate (Virusys Corporation, USA) and CEF peptide pool (Pantecs GMBH, Germany); which contains 32 peptides derived from cytomegalovirus, Epstein Bar and Influenza viruses; were used as positive controls for antigen-specific responses. Staphylococcal enterotoxin B (SEB; Sigma, MO) was used as a polyclonal positive control for ELISpot and intracellular staining assays.

### Interferon-γ ELISpot assay

Approximately 15 ml of whole blood were collected into EDTA vacutainer tubes (BD Biosciences, USA). Peripheral blood mononuclear cells (PBMC) were isolated by Ficoll-Hypaque density gradient centrifugation and viability was determined by trypan blue exclusion. The ELISpot assay was performed as described [24]–[26]. Briefly, PBMC (2×10^5^ cells/well) were incubated in triplicate cultures in the ELISpot plates (Whatman Unifilter, USA) coated with anti-human IFNγ antibody (MabTech, Sweden) and incubated for 16 hours with or without recombinant HCV antigens at 3 µg/ml of each single peptide in complete RPMI-1640 medium. Negative and positive controls included medium containing DMSO alone and Cytomegalovirus lysate, CEF peptide pool and 0.1 µg/ml SEB or other polyclonal stimuli, respectively. At the end of the incubation period, the assay was developed until the appearance of spots. The wells were, then, rinsed with tap water to stop the reaction. The number of spots per well was counted using an automated ELISpot reader (Cellular Technology Ltd., USA). Mean numbers of IFNγ spot forming cells (SFC) in control wells were subtracted from antigen-stimulated wells to correct for background cytokine production and are expressed per 10^6^ PBMC. An HCV antigen-specific response was considered positive if the number of SFC in the presence of antigen was at least 3-fold the number of SFC in the medium control, there are more than 5 spots per well and if there were ≥55 SFC/million PBMC [24], [26]–[28].

### Flow cytometric analysis of intracellular IFNγ by HCV-specific T cells

PBMCs were stimulated with HCV antigens and the intracellular cytokine production was examined as described previously [29]. Briefly, one million cells in complete RPMI 1640 medium were stimulated with the indicated HCV peptide pools at 3 µg/ml in a short-term assay of 16 h. Brefeldin A (10 µg/ml) was used during the last 15 h of the assay to inhibit cytokine secretion. Negative and positive controls were medium alone and SEB at 5 µg/ml, respectively. After the stimulation period, PBMC were washed and stained with surface fluorescein isothiocyanate (FITC)-labeled anti-human CD4 (BD Biosciences), and PerCP anti-human CD3 (BD Biosciences, CA, USA) at 4°C for 30 min. The cells were then washed, fixed, and permeablized for 10 min with FACS Perm 2 solution (BD Biosciences). Then, the cells were washed and stained with APC-labeled anti-human IFNγ MAb (BD Pharmingen) for 30 min at room temperature in a dark place. The cells were then washed, fixed, and stored until data acquisition on a FACS Calibur flow cytometer (BD Biosciences). Approximately, 100,000 total events were acquired and cytokine-producing cells were analyzed using the FlowJo software (Tree Star Inc., CA). The cells were gated on small lymphocytes using side and forward light scatter profiles and then on CD3^+^ T cells. IFNγ synthesis was shown as a function of CD4 expression on gated CD3^+^ T cells. The percentage of CD4^+^ and CD4^-^ T cells (mostly CD8 T cells) producing IFNγ was determined. Negative controls and compensation tubes were used to verify the assay and staining specificity.

### Statistical analysis

All data were entered into a Microsoft Access database (Microsoft Corp., USA). Duplicate data entry was performed to ensure quality control. Analysis was done on SPSS package version 17.0. (SPSS Inc., USA). Chi-square test was performed for categorical data, while student's *t*-test (or Mann Whitney U test when appropriate) was performed for comparison of continuous data. Correlation between different parameters was performed using Pearson's rank test. A *p* value of 0.05 or less was considered significant.

## Results

### HCV-specific cell-mediated immune responses to GT-1b and GT-4a peptides were similar

The demographic and laboratory characteristics of the study subjects (35 HCV infected/exposed subjects and 10 healthy controls) are shown in Table (1). There was no significant difference in any of the parameters tested between the responding and non responding subjects (as defined in the Subjects and Methods section). Among the 35 studied subjects, serum HCV-RNA was positive in 21 and 14 subjects were negative (below detection limit). All the 21 HCV-RNA positive subjects were confirmed to be infected with HCV GT-4 [5]. The 14 RNA negative subjects were assumed to have had GT-4 infection as it represents >90% of HCV infections in Egypt [5], [30], [31]. Seventeen (seven HCV-RNA negative and ten HCV-RNA positive) out of the 35 HCV antibody positive subjects (48.6%) responded to at least one of the 14 antigenic peptide pools (responders) while eighteen were non-responders. Among the 17 responders, 13 subjects (76.5%) had positive IFNγ production to both GT-1b and GT-4a peptide pools while 4 subjects (23.5%) only reacted to either GT-1b or GT-4a antigens (two subjects each). The mean (±SEM) total SFC (for positive responses only) in response to the seven peptide pools among the responding subjects was 216±55.8 and 199±55/10^6^ PBMC when cultured with GT-1b and GT-4a peptides, respectively (*p* = 0.833). The mean SFC in the non-responding subjects was 33±18 and 40±11.2 when cultured with GT-1b and GT-4a, respectively. The average SFC/10^6^ PBMC among chronic HCV responding subjects was 177±36.6 and 179±64.7 for genotype 1b and 4a, respectively while this was 271±128.3 and 228±121.6 among the resolved subjects for both genotypes, respectively. There was no significant differences between resolved and chronic subjects using GT-1b or 4a antigens (*p*>0.05). On the other hand, only one healthy control subject out of 10 subjects tested had a positive response to only one peptide pool (pool H genotype 4a) with only 137 SFC/10^6^ PBMC.

**Table 1:** Demographic, clinical and laboratory characteristics of the study subjects.

		HCV exposed subjects				Healthy Controls (n = 10; %)
Item	Total (n = 35) n(%)	Responders (n = 17; 48.6%)		Non Responders (n = 18; 51.4%)	*P* value	
**Female**	11(31)	5(29)		6(33)	0.809	8(80)
**Age (years)** *	41.5±11.3	39.87±10.16		41.31±10.62	0.442	33±8.05
**Rural Residence**	26(74)	12(70)		14(78)	0.638	7(70)
**Occupational Category**						
**-Medic**	3(8.6)	3(18)		0(0)		0
**-Nursing staff/student**	6(17.1)	3(18)		3(16.7)	0.305	0
**-Lab technician**	1(2.8)	1(6)		0(0)		0
**-Housekeeping**	17(48.6)	5(29)		12(66.7)		6(60)
**-Others**	8(22.8)	5(29)		3(16.7)		4(40)
**ALT level (IU/L)**	34.3±20.3	39.9±31.4		40.66±31	0.782	37.6±3.6
**HBsAg**	0(0)	0(0)		0(0)	NA	0(0)
**HCV RNA positive: n(%)**	21(60%)	10(58.8%)		11(61%)	0.836	0(0)
**Viral load (IUx10^3^/ml±SE)**	4,411±2,268	7,537±4,493		1,569±729	0.1	0(0)

The subjects were classified as responders and non responders as defined in the Subjects and Methods section. Specifically, responders and non-responders are those individuals who elicited or failed to elicit a positive HCV-specific IFNγ response in the ELISpot assay to one to fourteen HCV G-1b, and 4a overlapping 15-mer peptide pools, respectively. Ten healthy subjects with no known exposure to HCV served as a control group.

*Mean ± SD.

### Both T cell subsets produce IFNγ in response to stimulation with HCV GT-1b and 4a peptides

**Figure 1:**
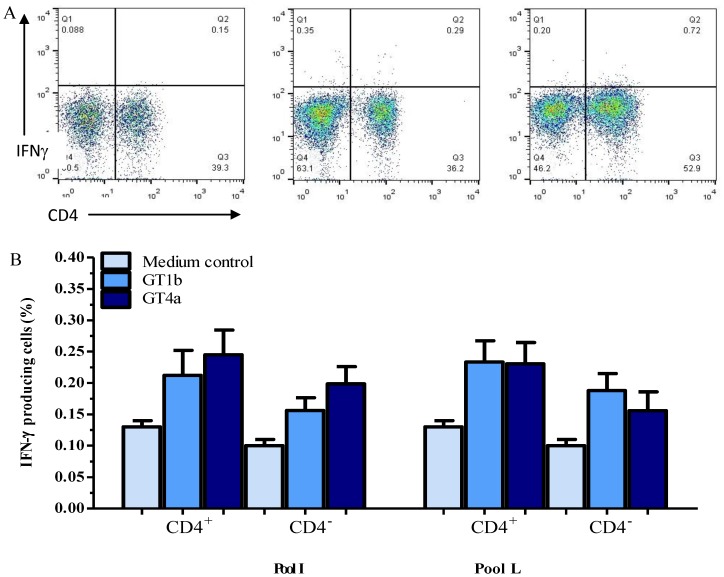
Flow cytometric analysis of cells responding to HCV peptide antigens derived from GT-1b and 4a shows capability of both CD4^+^ and CD4^-^ T cells to produce IFNγ. PBMC were stimulated with two sets of seven pooled overlapping 15-mer HCV GT-1b, and 4a peptide antigens as described in the Subjects and Methods section. The cells were gated on small lymphocytes using side and forward scatter profiles and then on CD3^+^ cells (not shown). **Panel** (**A**) shows an example of the production of IFNγ by CD4^+^ and CD4^-^ T cells in the presence of negative control (DMSO containing medium, left of panel A) and responding antigen pool L from both GT-1b (right panel A) and 4a (middle of panel A). **Panel B** shows the cumulative percentage of CD4^+^ and CD4^-^ T cells producing IFNγin response to HCV antigen pool I (NS5a) and L (N terminal NS5b) from GT-1b and 4a among Egyptians with chronic (n = 8) or resolved (n = 4) GT-4 HCV infection. Error bars represent the SEM.

Intracellular staining for IFNγ showed that both CD4^+^ and CD4^-^ T cells were capable of synthesizing this cytokine in response to both sets of pooled peptides and the findings were in agreement with the ELISpot results in magnitude and breadth. In this regard, an example of intracellular staining is shown in Figure 1A for CD3-gated (T) cells and illustrates that IFNγ was synthesized by both CD4^+^ and CD4^-^ T cells by PBMC stimulated with Pool L (N terminal NS5b) of both genotypes. The cumulative percentages of IFNγ producing CD4^+^ and CD4^-^ T cells for the HCV GT-1b and 4a pooled peptides I (NS5a) and L in eight chronic, and four resolved HCV subjects are shown in Figure 1B. As shown, the average percentage (±SE) of IFNγ producing CD4^+^ T cells in response to stimulation with pools L peptides derived from genotype 1b and 4a was 0.23±0.03 and 0.23±0.03, respectively. On the other hand, the average percentage of IFNγ producing CD4^-^ T cells in response to stimulation with pool L peptides derived from genotype 1b and 4a was 0.19±0.03 and 0.16±0.03, respectively. Similar data were obtained with pool I (Figure 1B) and other antigen pools. No significant differences were found between GT-1b and 4a antigens in both CD4^+^ and CD4^-^ T cells or between the percentage of T cell subsets producing IFNγ (*p*>0.05).

### Breadth of CMI response upon stimulation with peptides derived from both GT-1b and 4a

The 17 subjects who responded to at least one HCV antigen pool from the two overlapping peptide antigen sets derived from both GT-1b and GT-4a are shown in Table 2 with an average response to 1.65±0.24 and 1.65±0.25 antigen pools, respectively. No significant differences were found in the CMI responses to both peptide sets (*p* = 1). Data are shown in Table 2 as the mean IFNγ SFC/10^6^ PBMCs for each individual pool against the two sets of HCV antigens derived from both GT-1b and 4a in the responding subjects. As shown, the highest IFNγ production from PBMC of responding subjects for both GT-1b and GT-4a peptides was in pool F (NS3) and pool I (NS5a), respectively (Table 2). However, there were no significant differences between the two genotypes in the responses against any of the pools tested (*p*>0.05).

**Table 2:** Breadth of CMI response among HCV-responders to peptides derived from GT-1b and GT-4a.

Peptide pool	GT-1b SFC (mean±SE)	GT-4a SFC (mean±SE)	*p* value	Correlation Coefficient (r)
**E2 (Envelope)**	42±10	64±40	0.65	−0.089
**F(N terminal half of NS3)**	85±46	30±8	0.25	0.308
**G (remaining half of NS3)**	63±18.7	36±9	0.20	−0.059
**H (NS4a and NS4b)**	29±6.9	36±9.5	0.53	0.616**
**I (NS5a protein)**	32±14.7	74±29	0.20	0.897**
**L (first half of NS5b)**	37±6.8	51±5.6	0.15	0.406
**M (remainder of NS5b)**	35±8.9	46±7.9	0.40	0.359

PBMC were stimulated in triplicates with two sets of seven pooled overlapping 15-mer HCV genotype GT-1b, and 4a peptide antigens (E2 through M) for 16h as described in the Subjects and Methods section. The average number of HCV-specific SFC for the individual peptide pools (E2 through M±SEM) in the responding subjects is recorded for each pool and is shown for both genotypes. The responses shown are after subtraction of the DMSO control. The correlation of the CMI response to both HCV antigen sets in the different HCV proteins is also shown.

**High correlation.

### Correlation of CMI responses to GT-1b and GT-4a antigens among the 17 responders

**Figure 2:**
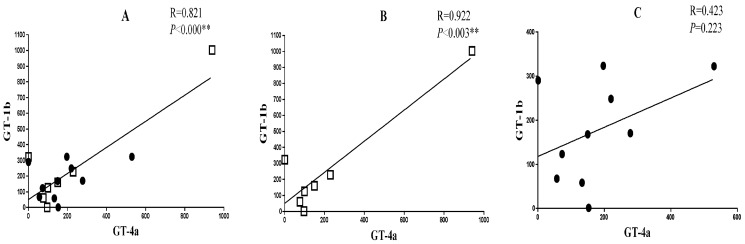
High correlation between cell-mediated immune response to HCV-pooled peptides derived from genotypes 1b and 4a. PBMC isolated from Egyptians with chronic or resolved HCV infection were stimulated in triplicates with two sets of seven pooled overlapping 15-mer HCV genotype 1b, and genotype 4a peptide antigens (E2 through M) for 16 h as described in the Subjects and Methods section. The total IFNγSFC recorded for each pool is shown for both genotypes. The total number of SFC for all responding donors to GT-4a pools was correlated with those from GT-1b in **Panel** (**A**) while that of resolved and chronic subjects are shown in **Panels** (**B**) and (**C**), respectively.

To assess the relationship between the CMI responses to the peptide antigens derived from both genotypes among genotype 4 infected/exposed subjects, the total SFC of responders to GT-1b antigens was correlated with that of GT-4a. This correlation was very strong (r = 0.82, *p*<0.001; 95% confidence interval [CI] = 0.562–0.933) in the 17 responders (Figure 2A). It was greater in the seven subjects who were HCV-RNA negative (r = 0.92; *p* = 0.003; CI = 0.551–0.989; Figure 2B) than in the 10 who had chronic HCV infections (r = 0.42; *p* = 0.022; CI = −0.282–0.831; Figure 2C). Also, as shown in Table 2, the highest correlation between the two genotypes in the different gene segments was in pools H (NS4a and NS4b; r = 0.616, CI = 0.133–763; *p* = 0.008) and pool I (NS5a; r = 0.897, CI = 0.329–0.574; *p*<0.0001) while a weaker correlation was found in the remaining pools (Table 2).

## Discussion

This study was conducted to assess whether Egyptians and others exposed to HCV GT-4 infection can benefit from HCV GT-1-based vaccines. We showed that seventeen of 35 subjects (48%) responded to at least one peptide pool derived from HCV GT-1b and/or GT-4a. No significant differences were found in the CMI responses to GT-1b and GT-4a antigens at different HCV gene segments or in the breadth of the response. Also, there was no significant difference in the responses of the seven subjects who had cleared their HCV infection and those 10 who went on to a chronic hepatitis C course. A strong correlation was found in the CMI responses to both HCV genotype peptides (r = 0.821, p<0.001; 95% CI = 0.562–0.933).

GT-4 is the predominant HCV genotype among Egyptians [5], [11], [30], [31], while the majority of HCV candidate vaccines that are currently under trials in Europe and USA are based on GT-1b. In this study, we compared the immune response of Egyptians infected with HCV GT-4 to antigens derived from both GT-1b and GT-4a as a marker to the effectiveness of an HCV-1b vaccine in Egypt and other areas where G-4 predominates. We assumed that the resolved subjects had HCV genotype 4 infections based on a well established body of evidence that GT-4 is the predominant genotype in Egypt. The accumulated data in the literature [5], [11], [30], [31] show that genotype 4 represents ∼90% of HCV genotypes in Egypt and suggest that; at the maximum; one of the seven responding subjects would have a genotype other than 4.

There are very limited data comparing the CMI response of humans to different HCV genotypes. A comprehensive analysis of cross-genotype reactivity of CD8^+^ T cells specific for one of the most frequently recognized HCV T cell epitopes has shown that T cells specific for wild-type GT-1 NS3_1073_ peptide, also, showed activity against HCV genotypes 4, 5 and 6 but not against genotypes 2 and 3 [32]. The authors highlighted significant inter-individual differences in response patterns, e.g., one individual that had recovered from GT-1 mounted a particular strong response against a single genotype 2 variant, which they related to previous exposure to HCV GT-2 [32] or differences in the specificity of cross-reactive memory T cells [33]. Also, chimpanzees previously infected with HCV when challenged with a homologous HCV genotype were frequently protected from infection while exposure to heterologous genotypes usually led to viremia [34]. When chimpanzees were re-exposed to a heterologous HCV strain, they were usually viremic for a longer time and some developed chronic infections [35]. However, previous experience with a putative HCV-1b vaccine showed cross protection against heterologous challenges [21]. Also, Barnes and colleagues showed that GT-1b vaccine-induced cross reactivity to GT-1a and GT-3a antigens [20] suggesting that Egyptians and others exposed to GT-4 infection could benefit from a GT-1b vaccine.

The concept of the presence of cross reactive CMI responses between HCV genotypes has been proved by vaccine/challenge experiments among four chimpanzees [36]. The authors suggested that epitopes conserved between genotypes must play an essential role in cross genotype immunity and protective immunity was often associated with an early increase in gamma interferon transcripts in the liver [36]. This concept was further confirmed among four other chimpanzees [37]. The evaluation of CD8 T cells among chronic HCV subjects; infected with genotypes other than genotype 1a; showed cross reactivity against HCV genotype 1a core antigen, which is relatively conserved between genotypes [38]. The data presented herein are in agreement with this notion. Importantly, our study is a comprehensive analysis of HCV-specific CMI responses comparing the responses of genotype 4-exposed subjects to almost all HCV antigens (E2 and all the non structural proteins). In this regard, there are very few data; if any; comparing the CMI responses of humans to different HCV genotypes.

Studies measuring CMI responses to HCV GT-4 among HCV-infected subjects used either recombinant proteins derived from GT-1 [39], [40], G-4a [41], or a restricted set of GT-1 [42], GT-4 [25] or a large set of GT-4a peptides [24]. Here, we had the opportunity to compare the HCV-specific CMI response using recombinant HCV pooled peptides derived from GT-1b and GT-4a; which covers almost the whole HCV proteins. Our results are similar to other reports of immune response to HCV [41], [43]. We did not find any significant difference in the response of patient infected with HCV GT-4; or those who had spontaneously-resolved the infection; to peptides derived from either GT-1b or GT-4a in any of the different gene segments. In this regard, the focus of this study was the comparison of CMI responses to HCV peptides derived from two different genotypes to examine whether patients infected with genotype 4 could mount a response to genotype 1 antigens. We wanted to have a considerable number of CMI responding subjects regardless of their HCV status. The fact that there was a difference but not significant between chronic and resolved subjects in this study may be attributable to the fact that this study was a cross sectional study with a small number of subjects in each category. Also, the time of HCV infection or resolution was not known for both chronic and resolved subjects. Earlier studies showed that the strength and magnitude of the HCV-specific CMI response are related to the time post infection for both chronic and resolved subjects [21], [44].

The responses in this study were limited in breadth in many of the subjects with the average number of responding pooled peptides for both GT-1b and GT-4a being the same. Also, there were no significant differences in the total number of IFNγ SFC between both genotypes and even in the response to different HCV proteins. Others have, also, reported that HCV-specific CMI responses were weaker among those having chronic HCV [45] and decline in those resolving HCV infection [45], [46]. In our study, only one of the 10 healthy controls who had no known exposure to HCV had a positive response to only one peptide pool (pool H genotype 4a).

Our results show that upon stimulation with peptide pools either from GT-1b or GT-4a, production of IFNγ by subjects infected with HCV genotype-4a is very similar and had a strong correlation. This correlation was greater among those who have cleared their HCV infections than in those with persistent viremia. The strongest correlation was in the peptide pools containing NS4a, NS4b and NS5a. Our results suggest that Egyptians or those potentially exposed to HCV GT-4 could be protected by an effective HCV vaccine derived from HCV GT-1. The data suggests that cross-strain recognition is possible. One of the short comings of this study is that we used pooled peptides just to screen for the cross match and correlation of HCV-specific response between both GT-1b and GT-4a among Egyptians who were infected with/or had spontaneously resolved HCV GT-4. We did not look at the fine mapping of the responses at the individual peptide level to determine the exact epitopes responding in each pool in both genotypes; which could be examined in future studies.

In summary, a strong correlation was found in the CMI responses to both GT-1b and GT-4a antigens among Egyptians exposed to HCV infection; whether with chronic or resolved infection. The data accumulated in the present study are parallel to prior studies that prove the presence of cross reactive CMI response between HCV genotype 1 and 4. These data suggest that Egyptians; as well as those exposed to GT-4; could benefit from a GT-1b vaccine. In conclusion, we believe that our *ex vivo* comparative findings have significant consequences for the development of an HCV vaccine that is effective against heterologous genotypes.

## Supporting Information

Table S1 **Raw data and characteristics of the study subjects and controls (demographic, clinical history and laboratory characteristics).** The subjects' are sorted according to category and their CMI response. Codes are shown at the bottom of the table.
(PDF)

Table S2 **Raw data of IFNγ HCV-specific immune response upon stimulation of PBMCs with 14 HCV genotype 1b and 4a overlapping peptide pools (seven pools from each genotype) as described in the Subjects and Methods section.** The subjects are sorted according to CMI response and HCV category. Positive (SEB and CMV) and negative (culture medium with DMSO) controls are, also, shown.
(PDF)
