# Supplementary material for: Cross Reactive Cellular Immune Response to HCV Genotype 1 and 4 Antigens among Genotype 4 Exposed Subjects
Source: PLoS One. 2014 Jun 30;9(6):e101264. doi: 10.1371/journal.pone.0101264 (PMC4076338; doi:10.1371/journal.pone.0101264)
Supplement: Table S1 — Raw data and characteristics of the study subjects and controls (demographic, clinical history and laboratory characteristics). The subjects' are sorted according to category and their CMI response. Codes are shown at the bottom of the table. (PDF) [file pone.0101264.s001.pdf]

Demographic data

#	PID	Gender	Age	Residence	Occupation	ALT	HCV-RNA titer IU/ml	anti-HCV ELISA average O.D	Category	HBsAg_ ELISA average O.D	Response in ELISPOT
1	120	1	45	1	5	21.0	0	1.10	1	0.11	Responder
2	197	1	40	1	3	54.1	0	2.37	1	0.26	Responder
3	242	1	35	1	4	33.2	0	2.81	1	0.11	Responder
4	667	2	38	1	2	17.5	0	3.34	1	0.11	Responder
5	669	1	52	1	5	10.5	0	1.40	1	0.12	Responder
6	322	1	49	1	1	26.2	0	2.31	1	0.10	Responder
7	705	1	25	1	4	17.5	0	3.20	1	0.11	Responder
8	101	1	31	0	4	41.9	0	3.17	1	0.12	non Responder
9	192	1	49	0	5	29.7	0	1.36	1	0.29	non Responder
10	419	1	27	1	4	22.7	0	3.38	1	0.07	non Responder
11	630	1	50	1	5	2.2	0	2.76	1	0.35	non Responder
12	337	1	50	1	4	29.7	0	3.40	1	0.10	non Responder
13	729	1	24	1	4	19.2	0	3.46	1	0.11	non Responder
14	346	2	34	1	2	12.2	0	3.45	1	0.10	non Responder
15	180	2	37	1	5	33.2	3350000	3.37	2	0.11	Responder
16	232	1	41	1	4	61.1	15600000	1.73	2	0.11	Responder
17	271	2	27	0	2	24.4	130000	3.18	2	0.12	Responder
18	300	1	57	0	1	143.2	38900	3.36	2	0.13	Responder
19	453	1	51	0	5	31.4	2640000	3.23	2	0.09	Responder
20	72	1	33	1	4	24.4	7230	off scale high	2	0.11	Responder
21	762	2	35	1	4	33.2	4470000	3.15	2	0.05	Responder
22	784	2	23	0	2	61.1	230000	3.16	2	0.10	Responder
23	813	1	28	1	5	41.9	907000	3.27	2	0.08	Responder
24	402	1	56	0	1	33.2	48000000	3.15	2	0.14	Responder
25	66	1	58	1	4	61.1	1150000	off scale high	2	0.11	non Responder
26	71	1	40	1	4	43.7	962000	off scale high	2	0.11	non Responder
27	99	1	53	1	4	21.0	1270000	3.35	2	0.11	non Responder
28	139	1	21	0	2	26.2	1570000	2.58	2	0.12	non Responder
29	166	2	57	1	4	40.2	37600	3.44	2	0.30	non Responder
30	190	2	45	1	5	64.6	2750000	2.45	2	0.71	non Responder
31	218	1	58	1	4	52.4	29200	1.50	2	0.10	non Responder
32	219	2	56	1	4	78.6	151000	1.21	2	0.74	non Responder
33	238	1	43	1	4	27.9	544000	1.95	2	0.11	non Responder
34	258	2	36	1	4	29.7	9670	3.20	2	0.14	non Responder
35	259	2	34	0	2	131.0	8790000	3.32	2	0.11	non Responder
36	260	1	38	1	4	24.4	0	0.19	3	0.11	Responder
37	94	1	25	1	4	40.1	0	0.33	3	0.11	non Responder
38	87	1	33	0	4	45.4	0	0.34	3	0.10	non Responder
39	131	1	27	1	4	15.7	0	0.08	3	0.14	non Responder
40	244	1	45	1	4	48.9	0	0.16	3	0.11	non Responder
41	246	1	48	1	5	19.2	0	0.16	3	0.11	non Responder
42	276	2	30	0	5	19.2	0	0.15	3	0.12	non Responder
43	395	1	33	0	5	26.2	0	0.26	3	0.11	non Responder
44	541	1	32	1	4	27.9	0	0.35	3	0.14	non Responder
45	633	2	21	1	5	26.2	0	0.36	3	0.12	non Responder
								Cut off > 0.8+mean of (-ve) control (<0.25)		Cut off=~ 0.160	

Code	Gender	Male	1	
		Female	2	
	Residence	Rural	1	
		Urban	0	
	Occupation	Medic	1	
		Nurse	2	
		Technician	3	
		Housekeeping	4	
		Other	5	
	Category	+VE/-VE	1	Resolved
		+VE/+VE	2	Chronic
		-VE/-VE	3	Control
