# Supplementary material for: Cross Reactive Cellular Immune Response to HCV Genotype 1 and 4 Antigens among Genotype 4 Exposed Subjects
Source: PLoS One. 2014 Jun 30;9(6):e101264. doi: 10.1371/journal.pone.0101264 (PMC4076338; doi:10.1371/journal.pone.0101264)
Supplement: Table S2 — Raw data of IFNγ HCV-specific immune response upon stimulation of PBMCs with 14 HCV genotype 1b and 4a overlapping peptide pools (seven pools from each genotype) as described in the Subjects and Methods section. The subjects are sorted according to CMI response and HCV category. Positive (SEB and CMV) and negative (culture medium with DMSO) controls are, also, shown. (PDF) [file pone.0101264.s002.pdf]

Responders 1-14 Antigen					Average of triplicate cultures/million PBMCs																	Total		total responding pools only	
	PaT ID	Group	Date	Plate #	CM	SEB*	CMV	E2-4	E2-1	F4	F1	G4	G1	H4	H1	I4	I1	L4	L1	M4	M1	G4	G1	G4	G1
1	197	+VE/-VE	25/7/2010	76	7	49993	30	1	0	83	102	52	37	88	68	58	32	38	58	37	40	358	337	230	228
2	242	+VE/-VE	2/8/2010	78	35	49965	50	688	0	70	798	0	127	15	0	95	0	53	78	88	0	1008	1003	940	1003
3	705	+VE/-VE	2/8/2010	79	0	50000	43	0	0	8	27	12	323	7	8	37	15	37	13	10	35	110	421	0	323
4	669	+VE/-VE	4/10/2010	91	0	100000	280	10	25	17	37	7	23	70	103	80	53	43	57	23	43	250	342	150	160
5	322	+VE/-VE	11/10/2010	93	18	99963	99963	0	23	97	0	27	20	0	7	13	0	27	0	7	0	170	0	97	0
6	667	+VE/-VE	4/4/2011	104	50	99950	1430	85	125	0	0	0	47	53	23	47	0	73	43	100	0	358	238	100	125
7	120	+VE/-VE	17/7/2011	118	7	99993	43	3	0	10	0	43	10	27	27	20	10	77	23	50	60	230	130	77	60
8	784	+VE/+VE	2/8/2010	78	8	49992	55	0	7	0	203	8	87	3	53	37	0	0	0	0	3	0	353	0	290
9	180	+VE/+VE	2/8/2010	79	23	50000	90	28	45	30	65	65	72	150	53	3	13	60	97	30	17	365	362	150	168
10	402	+VE/+VE	4/10/2010	91	2	99997	447	7	37	3	3	37	0	10	23	20	0	30	37	73	123	180	223	73	123
11	813	+VE/+VE	23/3/2011	100	17	99983	323	63	58	13	10	70	13	0	0	50	23	47	27	43	7	287	138	133	58
12	762	+VE/+VE	4/4/2011	105	7	99993	273	8	43	7	3	0	30	37	37	53	10	90	33	63	50	258	207	153	0
13	453	+VE/+VE	4/4/2011	105	13	99987	657	52	102	47	40	30	87	53	23	83	3	67	10	70	60	402	325	220	248
14	72	+VE/+VE	16/5/2011	111	27	99973	288	88	0	43	97	127	73	17	0	30	3	20	30	63	13	388	217	278	170
15	300	+VE/+VE	16/5/2011	112	47	99953	40	0	0	87	33	103	103	40	43	83	120	80	57	93	100	487	457	197	323
16	232	+VE/+VE	23/5/2011	113	17	99983	453	0	0	3	33	30	27	40	17	27	23	57	67	23	50	180	217	57	67
17	271	+VE/+VE	11/10/2010	93	3	99993	99993	47	85	0	0	7	0	5	0	530	237	12	7	2	0	602	328	530	322
		Average				*Usually too numerous to c		63	32	30	85	36	63	36	29	74	32	48	37	46	35	331	312	199	216
		SQRT						4.123	4.123	4.123	4.123	4.123	4.123	4.123	4.123	4.123	4.123	4.123	4.123	4.123	4.123	4.123	4.123	4.123	4.123
		SD						163.7	40	34	191	37	77	39	29	120	61	25	28	33	37	227	212	227	230
		SE						39.7	9.6	8.3	46.3	9.0	18.7	9.5	6.9	29.2	14.7	6.1	6.8	7.9	8.9	54.9	51.4	55.2	55.9
		Median						8	23	15	33	30	37	32	23	47	10	50	33	47	35	273	325	142	168

non Responders

	PaT ID	Group	Date	Plate #	CM	SEB	CMV	E2	E-2	F4	F1	G4	G1	H4	H1	I4	I1	L4	L1	M4	M1	T G4	T G1
1	630	+VE/-VE	25/7/2010	75	0	50000	90	18	8	0	0	0	0	7	0	3	7	27	5	28	2	83	21
2	101	+VE/-VE	25/7/2010	77	0	50000	30	0	0	0	0	0	0	0	0	0	0	0	0	0	0	0	0
3	729	+VE/-VE	11/7/2011	116	0	100000	170	5	0	3	10	3	0	10	3	3	3	3	0	3	3	32	20
4	419	+VE/-VE	12/7/2010	72	2	2288	156	13	18	15	8	7	0	48	0	38	0	5	0	0	5	127	32
5	346	+VE/-VE	4/10/2010	92	23	99953	1068	0	0	0	3	0	0	0	0	10	13	0	10	0	0	0	0
6	192	+VE/-VE	2/8/2011	120	17	99983	183	0	0	33	17	0	43	33	47	47	10	20	3	10	10	143	130
7	337	+VE/-VE	4/4/2011	104	257	99743	48	0	0	0	0	0	0	0	0	0	0	0	0	0	0	0	0
8	190	+VE/+VE	19/7/2010	73	0	5738	40	0	0	0	0	0	0	0	0	0	0	0	0	0	0	0	0
9	66	+VE/+VE	19/7/2010	73	0	50000	263	0	0	0	0	0	0	0	3	0	0	0	0	0	0	0	3
10	99	+VE/+VE	25/7/2010	75	0	50000	3	15	5	15	8	25	8	3	17	10	0	27	12	25	18	120	68
11	166	+VE/+VE	25/7/2010	76	7	49993	50	6	0	7	0	12	8	0	30	0	25	0	0	0	0	24	63
12	259	+VE/+VE	25/7/2010	77	0	50000	75	0	0	0	0	0	0	3	3	3	0	0	0	0	0	7	3
13	218	+VE/+VE	9/8/2010	83	0	50000	150	0	0	0	0	0	3	2	0	0	3	0	3	0	3	2	13
14	238	+VE/+VE	23/3/2011	101	0	100000	115	0	5	10	20	7	10	3	3	17	37	0	30	0	3	37	108
15	258	+VE/+VE	23/3/2011	101	5	99990	25	10	0	0	3	0	23	0	13	10	20	10	0	20	17	50	77
16	139	+VE/+VE	4/5/2011	109	7	99993	33	3	13	13	7	3	0	0	0	17	10	3	3	3	13	43	47
17	71	+VE/+VE	16/5/2011	111	27	99973	698	0	0	13	0	0	0	0	0	20	0	10	0	0	0	43	0
18	219	+VE/+VE	9/8/2010	83	0	50000	60	0	0	2	2	2	0	0	5	2	2	0	5	7	3	12	17

Healthy Control

	PaT ID	Group	Date	Plate #	CM	SEB	CMV	E2	E-2	F4	F1	G4	G1	H4	H1	I4	I1	L4	L1	M4	M1	T G4	T G1
1	276	-VE/-VE	17/7/2011	118	0	100000	30	0	0	10	13	3	3	0	17	10	13	13	0	17	0	53	47
2	94	-VE/-VE	28/7/2011	119	20	99980	2340	0	0	17	0	0	0	0	0	7	0	10	0	0	0	33	0
3	131	-VE/-VE	28/7/2011	119	0	1990	10	0	0	10	13	3	7	7	0	7	37	3	13	20	10	50	80
4	633	-VE/-VE	2/8/2011	120	13	3052	22	2	0	10	0	0	7	0	3	13	0	7	3	0	0	32	13
5	244	-VE/-VE	8/8/2011	121	7	99993	3	0	0	20	17	0	3	10	0	7	0	40	10	17	0	93	30
6	541	-VE/-VE	8/8/2011	121	0	2095	20	0	0	13	3	13	7	7	17	17	3	7	3	3	0	60	33
7	87	-VE/-VE	8/8/2011	122	7	3378	3	0	0	0	0	7	0	0	3	3	0	7	0	3	0	20	3
8	246	-VE/-VE	17/7/2011	117	20	99980	855	0	0	13	0	40	0	0	0	13	7	27	0	0	0	93	0
9	395	-VE/-VE	8/8/2011	122	0	100000	965	15	0	7	10	20	7	7	23	10	7	0	13	27	10	85	70
10	260	-VE/-VE	17/7/2011	117	7	2728	163	3	0	7	0	0	17	137	40	3	7	0	0	13	3	163	67
